# A comprehensive spectral assay library to quantify the *Escherichia coli* proteome by DIA/SWATH-MS

**DOI:** 10.1038/s41597-020-00724-7

**Published:** 2020-11-12

**Authors:** Mukul K. Midha, Ulrike Kusebauch, David Shteynberg, Charu Kapil, Samuel L. Bader, Panga Jaipal Reddy, David S. Campbell, Nitin S. Baliga, Robert L. Moritz

**Affiliations:** 1 Institute for Systems Biology, 401 Terry Ave N, Seattle, WA 98109 USA; 2 Departments of Biology and Microbiology, University of Washington, Seattle, WA USA; 3 Molecular and Cellular Biology Program, University of Washington, Seattle, WA USA; 4 Lawrence Berkeley National Lab, Berkeley, CA USA

**Keywords:** Proteomics, Proteome informatics

## Abstract

Data-Independent Acquisition (DIA) is a method to improve consistent identification and precise quantitation of peptides and proteins by mass spectrometry (MS). The targeted data analysis strategy in DIA relies on spectral assay libraries that are generally derived from *a priori* measurements of peptides for each species. Although *Escherichia coli* (*E. coli*) is among the best studied model organisms, so far there is no spectral assay library for the bacterium publicly available. Here, we generated a spectral assay library for 4,014 of the 4,389 annotated *E. coli* proteins using one- and two-dimensional fractionated samples, and ion mobility separation enabling deep proteome coverage. We demonstrate the utility of this high-quality library with robustness in quantitation of the *E. coli* proteome and with rapid-chromatography to enhance throughput by targeted DIA-MS. The spectral assay library supports the detection and quantification of 91.5% of all *E. coli* proteins at high-confidence with 56,182 proteotypic peptides, making it a valuable resource for the scientific community. Data and spectral libraries are available via ProteomeXchange (PXD020761, PXD020785) and SWATHAtlas (SAL00222-28).

## Abstract (summary)

**Table:** 

Measurement(s)	Proteome • database type spectral library
Technology Type(s)	SWATH MS protein profiling assay • mass spectrometry • Data-Independent Acquisition
Sample Characteristic - Organism	*Escherichia coli*

Machine-accessible metadata file describing the reported data: 10.6084/m9.figshare.13078733

## Background & Summary

Achieving systems-wide reliable identification and precise quantification of peptides and proteins remains a challenge for many organisms^1^. The most commonly used method to analyze the protein content of a biological sample is liquid chromatography-coupled tandem mass spectrometry (LC-MS/MS) operated in data-dependent acquisition (DDA) mode^1,2^. Due to the high dynamic range of proteins in complex proteomic samples and the stochastic nature of DDA, MS/MS sampling of precursor ions is biased towards more intense ion signals, limiting the consistent detection of low abundant peptides^3–5^. To overcome this bias and achieve broad proteome coverage, DDA is often combined with different fractionation techniques such as off-gel electrophoresis (OGE), high pH chromatography and ion exchange chromatography^1,6^ or paired with ion mobility gas-phase separation^7–9^. The expressed proteome for e.g., human*, Mycobacterium tuberculosis*, zebrafish and mouse was defined by applying such an experimental approach, resulting in the generation of deep proteome identification by DDA suitable for spectral assay library generation^6,10–12^.

While targeted MS methods including Selected Reaction Monitoring (SRM) and Parallel Reaction Monitoring (PRM) quantify predetermined sets of peptides that uniquely represent individual proteins across samples with a high degree of reproducibility, yet these methods are confined to quantify low numbers of peptides in a single injection^13,14^. DIA, also commonly known as Sequential Window Acquisition of all Theoretical spectra (SWATH), was introduced as an alternative data acquisition approach to comprehensively analyze and reproducibly quantify large fractions of a target proteome^15^. In DIA/SWATH-MS, preselected isolation mass windows are specified and fragmentation of all precursor ion species contained is performed in an efficient, unbiased manner, independent of their abundance^15–17^. This results in co-fragmentation of simultaneously eluting and close isobaric ion species and generates highly complex MS/MS spectra, which need to be deconvoluted by peptide-centric data analysis approaches^5,15,17,18^. In a peptide-centric analysis, a spectral ion library, also referred to as a SWATH or assay library, is used to extract ion chromatograms in a targeted manner^15,18^. Comprehensive, high-quality assay libraries support the identification and the consistent and accurate quantification of thousands of peptides representing large fractions of the proteome^16,17^. The reproducibility and quantitative performance of SWATH-MS has been assessed in an exemplary multi-laboratory study^19^. Routine quantification of complex proteomic samples requires a comprehensive spectral assay library and high-throughput, robust workflows, which can be accomplished by coupling DIA/SWATH-MS with faster chromatography^20,21^.

*E. coli* is an important model organism to study fundamental concepts in biochemistry and molecular biology, and is routinely used for industrial production of recombinant proteins^22–25^. In recent years, there have been significant contributions by several proteomic studies to understand the biology of *E. coli* using DDA-MS^26–30^. However, these studies failed to achieve comprehensive identification and quantification of the proteome in a single measurement or consistent quantification across many samples. To reach deep proteome coverage, these studies generally measured many experimental conditions and fractionated samples. In addition, these DDA based methods are expensive, time-intense, and technically complex, which limits their implementation for routine use across laboratories^6^. Therefore, through the application of state-of-the-art proteomic technologies including DDA-MS and multiple fractionation strategies, we have generated a comprehensive *E. coli* spectral assay library and implemented DIA/SWATH-MS workflows to facilitate routine quantification of the extractable *E. coli* proteome.

**Fig. 1:**
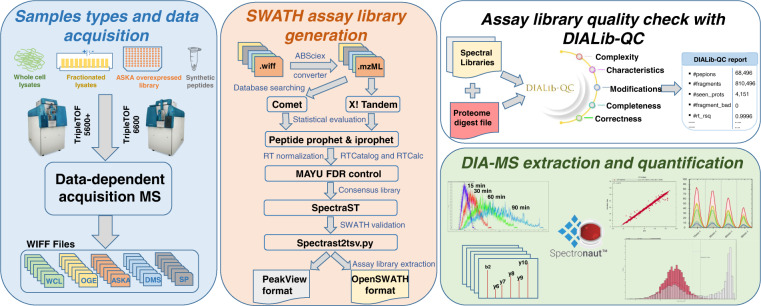
Data acquisition workflow to generate a comprehensive *E. coli* assay library, quality evaluation with DIALib-QC and DIA/SWATH-MS quantification by Spectronaut. A comprehensive DIA/SWATH assay library for *E. coli* was generated from whole cell lysate, fractionated samples, overexpressed proteins, and supplemented with synthetic peptides. Samples were analyzed with data-dependent acquisition (DDA) mass spectrometry on TripleTOF 5600+ and TripleTOF 6600 instruments resulting in 209 data files. To generate a DIA/SWATH library, the raw data files were converted to mzML format using the ABSCIEX converter with the profile mode extraction parameter. The mzML files were searched against the reference proteome using both Comet and X!Tandem search engines. The identified sequences were then statistically validated using the Trans-Proteomic Pipeline (TPP) including PeptideProphet and iProphet. MAYU was applied to control the FDR at the protein level. Using SpectraST, confidently assigned spectra were converted into a redundant spectral library and retention times are normalized in iRT space using RTCatalog, then a consensus spectrum library was generated. The assay library was extracted from the consensus library using the spectrast2tsv.py script. Libraries were evaluated with the DIA Library Quality Control (DIALib-QC, www.swathatlas.org) tool and their assessment reports were generated. The performance of the TripleTOF *E. coli* spectral library was evaluated based on the identification and quantitation of peptides and proteins in data-independent acquisition (DIA) methods with different gradient lengths using the Spectronaut analysis software.

Here, we report a comprehensive, high-quality *E. coli* spectral assay library for the quantification of 56,182 proteotypic peptides mapping to 4,014 *E. coli* proteins representing 91.5% of the annotated proteome. Considering 690 additional peptides that are shared between proteins, 4,086 proteins can be measured with this spectral assay library (93.1% of the proteome). The library was generated from 209 measurements of unfractionated and fractionated *E. coli* cell lysates using OGE and differential ion mobility (DMS), overexpressed proteins, and synthetic peptides enabling deep proteome coverage on SCIEX TripleTOF 5600+ and 6600 instruments. This library has been statistically validated with MAYU^31^ and its quality assessed with the spectral library tool DIALib-QC^32^ (www.swathatlas.org) (Fig. 1). The *E. coli* spectral assay library is transferrable to other instruments collecting DIA data, is a valuable resource for the scientific community, and is publicly available at SWATHAtlas (SAL 00222-28) and ProteomeXchange (PXD020785^33^). We demonstrate the utility of this spectral assay library to consistently quantify the *E. coli* proteome with minimal technical variability and rapid-chromatography workflows accelerating data acquisition up to 4-fold for increased sample throughput.

## Methods

### Sample overview

To achieve a comprehensive representation of the *E. coli* proteome we analyzed samples from total cell lysates, overexpressed proteins, and selected synthetic peptides representing proteins not detected from the before mentioned sources. Samples were analyzed as unfractionated samples, by off-gel electrophoresis and differential mobility separation providing orthogonal separation of analytes in combination with LC-MS/MS analysis. The specific samples and analysis conditions are summarized in Table 1. Figure 1 illustrates the experimental workflow for DIA/SWATH assay library generation, the evaluation of the quality of the assay library with the DIA Library-Quality Control (DIALib-QC) software (www.swathatlas.org), and shows the performance of the library with different gradients measured in DIA/SWATH mode.

**Table 1:** Sample overview.

Sample Type	Peptide fractionation	Medium	Instrument	MS injections
**ASKA(-) overexpressed library**	None	LB	TT5600 +	58
**Whole cell lysate (WCL)**	None	LB/M9	TT5600+, TT6600	33
**Whole cell lysate (WCL)**	OGE	LB	TT5600+	24
**Whole cell lysate (WCL)**	OGE	M9	TT5600+	24
**Whole cell lysate (WCL)**	DMS	M9	TT5600+	47
**Synthetic peptides**	None	None	TT5600+	23
**Total**				209

Sample types including peptide fractionation method, MS instruments and number of injections that were used to generate the *E. coli* spectral library are depicted. ASKA (-) refers to overexpressed strains with histidine-tagged proteins, OGE: Off gel electrophoresis, DMS: differential ion mobility, LB: Luria-Bertani broth medium, and M9: Minimal medium.

### *E. coli* strains and growth conditions

All experiments reported in this study were performed with lysates from an *E. coli* K12 strain and a set of *E. coli* open reading frame (ORF) archive strains devoid of green fluorescent protein (ASKA(-) library Host Cell AG1(ME5305)) that overexpress individual ORFs fused to a histidine-tagged protein^34^, and was purchased from the National Institute of Genetics (NIG), Microbial Physiology Laboratory, (NBRP), 1111 Yata, Mishima, Shizuoka, 411-8540 JAPAN (https://shigen.nig.ac.jp/ecoli/strain/). All strains were grown in Luria-Bertani broth (LB) medium (yeast extract (Becton and Dickinson (BD)), Tryptone (BD) and sodium chloride (Millipore-Sigma)), or M9 medium (47.8 mM Na_2_HPO_4_ (BDH/VWR), 22 mM KH_2_PO_4_ (Fluka), 8.6 mM NaCl (Fluka), 18.7 mM NH_4_Cl (Fisher Scientific)).

### Whole cell lysate sample preparation

Cell pellets from *E. coli* whole cell lysates (WCL) were resuspended in 2 mL 8 M Urea (VWR, USA) and 50 mM NH_3_HCO_4_ (AmBic) (Millipore-Sigma) per gram of wet weight pellet, sonicated 6 × 15 s, output level 2, 40%, 45 s on ice with a sonifier 250 (Branson). Insoluble cell debris was precipitated by centrifuging the sample for 10 min at 16,000 x g at 4 °C. Protein concentration was determined by BCA protein assay (Pierce). Proteins were reduced with 10 mM DTT (Millipore-Sigma) at 56 °C for 25 min and alkylated with 14 mM Iodoacetamide (Millipore-Sigma) for 30 min in the dark. Proteins were digested with sequencing grade Trypsin-Gold (Promega) and a protein to enzyme ratio of 1:100 overnight at 37 °C. Digestion was stopped by lowering the pH to 2.0 and peptides desalted using C18 SepPak columns (Waters) following the manufacturer’s protocol.

### Growth of ASKA(-) *E. coli* overexpressed green fluorescent protein negative strains

2 mL LB medium were inoculated with each single ASKA (-) Host Cell AG1(ME5305) strain required to build the spectrum ion library and incubated overnight at 37 °C. To increase throughput and simplify sample processing, we combined pre-cultures from multiple single ORF overexpression strains (between 28 to 96 strains) in a single culture and diluted with 500 mL pre-warmed LB medium. After 1-hour growth at 37 °C, protein expression was induced with 1 mM Isopropyl β- d-1-thiogalactopyranoside (IPTG). Cells were harvested after 4 hours by centrifugation at 4 °C for 20 min at 25,000 x g, which resulted in a cell pellet and accumulated expressed protein as insoluble aggregates or ‘inclusion bodies’. After selective urea washing of the inclusion bodies, to remove extraneous proteins, inclusion bodies were resolubilized in 8 M guanidinium hydrochloride^35^. Proteins were reduced and alkylated as described for the whole cell lysate above. Buffer was exchanged with 1% AmBic by size exclusion chromatography (PD-10, GE Healthcare) before enzymatic digestion overnight with trypsin at 37 °C.

### Off gel electrophoresis fractionation

To maximize the proteome coverage from whole cell lysates, the samples grown in both LB and M9 medium were fractionated by off-gel electrophoresis (OGE). After digestion and desalting, 100 µg of whole cell lysate derived peptides were re-solubilized in OGE buffer (5% (v/v) glycerol, 0.7% acetonitrile, 1% (v/v) carrier ampholytes mixture IPG buffer pH 3–10, GE Healthcare). Peptides were separated on a 3100 OFFGEL Fractionator (Agilent Technologies) using an immobilized pH gradient strip pH 3–10, 24 cm (GE Healthcare) at a maximum of 8,000 V and 50 μA until 50 kVhrs were reached. 24 in-solution fractions (from each medium) were acidified to pH < 2 with trifluoroacetic acid, individually desalted (Sep-Pak tC18 96-well μ-elution plate, Waters) and vacuum dried (Savant) prior to DDA LC-MS/MS.

### Synthetic proteotypic peptides

For proteins not detected in *E. coli* WCL or not amenable to overexpression via the growth of ASKA(-) strains described above, we selected, as far as possible, proteotypic peptides to increase the proteome coverage following the criteria described in Kusebauch *et al*.^36^. For a few peptides, the selection criteria were relaxed (e.g., hydrophobicity) as otherwise the respective proteins would be excluded *a priori*. Likewise, for protein identifications based on a single peptide, we selected an additional synthetic proteotypic peptide. 1,358 peptides covering 742 *E. coli* proteins were individually chemically synthesized as free amine at the N-terminus and carboxylic acid at the C-terminus, cysteine residues were incorporated as carboxyamidomethylated cysteine building blocks (PEPotec SRM library Grade 1, Thermo-Fisher Scientific). Peptides were acquired in pools of up to 95 peptides and diluted to 5% acetonitrile, 0.1% formic acid in water (v/v).

### Data dependent acquisition (DDA) mass spectrometry for spectral assay library generation

DDA was performed on both a TripleTOF 5600+ (SCIEX) and a TripleTOF 6600 mass spectrometer (SCIEX), both interfaced with a micro-LC interfacePlus HPLC system (Eksigent) configured in either nano-flow or micro-flow mode. Both systems were operated with 99.9% water, 0.1% formic acid (buffer A) and 99.9% acetonitrile, 0.1% formic acid (buffer B). For the chromatographic separation in nano-flow mode, peptides were trapped on a C18 trap column, 3 µm, 120 Å, 20 mm × 75 µm (Dr. Maisch) at 2 µL/min for 10 minutes and separated on a C18 separation column, 3 µm, 120 Å, 150 mm × 75 µm (Dr. Maisch) at 300 nL/min. Three different gradient lengths were used based on the complexity of the samples **(**Supplementary Table 1**)**. For the separation in micro-flow mode, peptides were loaded on a 10 mm × 300 µm trap cartridge Chrom XP C18CL, 5 µm, 120 Å (Eksigent) at 10 µL/min for 10 minutes and separated on a ReproSil-Pur C18-AQ, 2.4 µm column, 15 cm × 200 µm (Dr. Maisch GmbH) at 5 µL/min using a 90-minute gradient **(**Supplementary Table 1**)**. For the nano-flow measurements, 10 µm ± 1 µm silica tip electrospray emitters (New Objective, P/N FS360-20-10-N-20-C12) with no coating were used and ion source parameters were as follows: ISVF = 2,300, GS1 = 3, GS2 = 0, CUR = 25, TEM = 150, while for micro-flow, the ion source was equipped with a 25 µm Turbo Ion Spray probe (SCIEX) and parameters were set to: ISVF = 5,500, GS1 = 15, GS2 = 15, CUR = 25, TEM = 100.

DDA replicates in top-10 and top-20 mode configuration were acquired on Triple TOF 5600 + and 6600 instruments **(**Table 1**)**. MS1 spectra were collected in the range 400–1,250 *m/z* for 250 milliseconds (ms) accumulation time and fragment ion spectra were collected in the range of 100–2,000 *m/z* for 150 ms accumulation time. The selected precursors were then added to a dynamic exclusion list of 20 s. Rolling collision energy with a collision energy spread of +/− 5 V was used for fragmentation to mimic SWATH like fragmentation conditions.

### SelexION Differential Mobility Separation (DMS)

Further, *E. coli* samples grown in M9 medium (Table 1) were fractionated by DMS^9^. The SelexION device was operated with a dispersion voltage of 3,800 V on a TripleTOF 5600+, the temperature was set to medium (225 °C). Source conditions and compensation voltages (CV) were optimized using a standard peptide at 15 V. The whole cell lysate sample was injected repeatedly with different CV values scanning from +4 to +27 V in 0.5 V steps, which resulted in total of 47 fractions. At each increment of CV, both MS and MS/MS spectra were recorded. The curtain and sheath gas were set to a flow of 25 and 6 arbitrary units, respectively, with throttle gas being disabled.

### Data Independent Acquisition (DIA) mass spectrometry

DIA was performed on a TripleTOF 6600 mass spectrometer (SCIEX), interfaced with a micro-LC interfacePlus HPLC system (Eksigent) configured in micro-flow mode. To demonstrate the performance of the assay library using the targeted data extraction strategy, data were acquired with four different gradient lengths **(**Supplementary Table 2**)**. The gradient flowrate, column and ion source conditions were used as described above for DDA micro-flow chromatography.

DIA/SWATH data were collected with an MS/MS ALL SWATH^TM^ Acquisition method using 100 variable acquisition windows^37^, each with a 1.0 Da overlap with the previous window. For each SWATH-MS duty cycle, an MS1 survey scan in high-resolution mode from 400 to 1,250 *m/z* and MS2 spectra in high-sensitivity mode from 100 to 1,500 *m/z* were collected with accumulation times based on the different gradient lengths **(**Supplementary Table 2**)**. This kept the total duty cycle time manageable from 1.7 seconds for the 15-minutes gradient to 3.3 seconds for the 90-minutes gradient. Five analytical replicates of each gradient of the *E. coli* cell digest were measured for statistical confidence, resulting in total 20 DIA/SWATH files.

### Spectral and assay library generation

In total 209 data-dependent acquisition raw mass spectrometry files stored in .wiff format **(**Table 1**)** were converted to profile mzML using the ABSCIEX MS data converter (version 1.3 beta). This converter has three modes, profile, centroid and protein pilot that can be selected. We explicitly used the profile mode for conversion of DDA.wiff files to mzML as its peak-picking algorithm outperforms other convertors tested. Comparing this converter with pwiz msconvert version 3.0.111220, the latter showed an inferior performance with TripleTOF data where less spectral scans were generated per file. The Trans-Proteomic Pipeline (TPP)^38^ (version 5.2.0 Flammagenitus) was used for the analysis of the shotgun proteomics runs. Spectra were searched with both Comet^39^ (version 2017.01) and X!Tandem^40^ (version 2013.06.15, native and k-score^41^) against the full non-redundant, canonical *E. coli* K12 reference proteome from UniProtKB/Swiss-Prot^42^ (Proteome ID UP000000625, November 2019) with 4,389 reviewed proteins and common contaminant proteins, decoy sequences and iRT peptides (Biognosys) appended. Carbamidomethyl (57.0214 Da) on cysteine was used as fixed modification and oxidation (15.9949 Da) on methionine was set as variable modification. Up to two missed tryptic cleavages were allowed. The precursor mass error was set to ±50 ppm, fragment bin tolerance was set to 0.05 *m/z*. The search identifications of these runs were combined and statistically scored using PeptideProphet^43^ and iProphet^44^ within the TPP^38^. MAYU^31^ (version 1.07) was used to select an iProphet cutoff of 0.99617, resulting in a protein false discovery rate (FDR) of 1%. A raw spectral library was built and filtered for a MAYU FDR of 1% using SpectraST^45^ in library generation mode with CID-QTOF settings for high resolution and high mass accuracy. A consensus library was consecutively generated according to Rosenberger *et al*.^6^ using spectrast2tsv.py (msproteomicstools 0.2.2; https://pypi.python.org/pypi/msproteomicstools).

### Retention time normalization

iRT peptides (Biognosys AG, Schlieren, Switzerland) were added to the *E. coli* whole cell lysate samples prior to MS injection according to vendor instructions^46^ for RT normalization across runs. Retention times of all *E. coli* peptides were indexed in iRT space for use in the *E. coli* spectrum library as follows: The RTCatalog tool, which is part of the TPP^38^, was applied iteratively in several phases to the *E. coli* peptide measurements. The initial run of RTCatalog was applied selectively to the data that included the iRT peptides (WCL data). This catalog was created by first transforming the retention times of all peptides to the iRT retention time index space, which was possible due to the presence of iRT peptides in each run. The second run of RTCatalog was applied to remaining data (except the synthetic peptide data) by referencing the common peptides of the initial WCL data catalog, bringing the RT of all *E. coli* peptides into iRT space. Thus, the second run of RTCatalog brought the retention times of all peptides in all the data from the non-synthetic runs, whether they were spiked with iRT peptides or not, into iRT space.

Finally, the retention times of the 1,358 synthetic peptides were theoretically computed in iRT space. We used, the RTCalc tool (also part of the TPP), where first, RTCalc was trained using the Linear Discriminant Model on the peptides and their median values from the *E. coli* RTcatalog described above. Secondly, the trained RTCalc model was employed to estimate the iRT values of the synthetic *E. coli* peptides to bring them to the same iRT space as the RTCatalog method above. The final list of retention times for the *E. coli* spectral library comprises the median iRT values of the second RTcatalog described above, comprising all but the synthetic peptides, appended with the theoretical RTCalc predictions for the synthetic peptides based on the RTCalc model trained on the same catalog.

### Spectral assay library quality control using DIALib-QC

Over 62 parameters of compliance of the assay library was evaluated using DIALib-QC^32^ (http://www.swathatlas.org/DIALibQC.php), a freely available tool that highlights the library’s complexity, characteristics, modifications, completeness and correctness **(**Fig. 1**)**. Assay libraries can also be evaluated online at https://db.systemsbiology.net/sbeams/cgi/PeptideAtlas/AssessDIALibrary. The DIALib-QC assessment report of the assay libraries for both PeakView and OpenSWATH format is provided in Supplementary Table 3.

### Validation and modification of assay libraries

Validating the assay library with the DIA/SWATH 100 variable Q1 isolation window scheme used for sample acquisition in DIA/SWATH mode, DIALib-QC reports 9,046 fragment ions that fall into the swath window of the precursor **(**Supplementary Table 3**)**. These fragment ions were excluded as the resulting signals can interfere with the quantitation. Next, the consensus library was reduced to the top 6 fragment ions of charge state 1 and 2 between 100 to 1500 *m/z* per precursor using spectrast2tsv.py from the msproteomicstools program as described above. Prior to DIA/SWATH analysis, contaminant and decoy assays were removed from the assay library. The SWATH 100 variable window validation, and modified library assessment report, is provided in Supplementary Table 3.

### DIA/SWATH data analysis

Spectronaut^47^ DIA software (version 13.11.200127.43655 (Laika), Biognosys) was used to perform the targeted data extraction of five analytical replicates of each gradient. For Spectronaut, the assay library was used directly as generated and described above. The HTRMS converter was used to convert the raw WIFF files into HTRMS files. For the nonlinear iRT calibration strategy, a dynamic window was used for both mass tolerance (MS1 and MS2), and to set up extracted ion chromatogram (XIC) RT window. Pre-processing of MS1 and MS2 calibration strategy was enabled. Decoy assays were dynamically generated using the scrambled decoy method with a set size of 1 as a fraction of the input library size. The identification was performed using the normal distribution estimator with precursor and protein identification results filtered with a q-value of <0.01. For quantification, MS2 ion peak areas of quantified peptides were summed to estimate the protein peak areas.

### Sample preparation quantitative SWATH-MS experiment

*E. coli* (ASKA library Host Cell AG1(ME5305) strain) was cultivated overnight from the glycerol stock in the presence of chloramphenicol (30 µg/mL) in three biological replicates. The following day, cultures were re-inoculated into fresh 200 mL LB media with initial optical density (OD) of 0.1 in the presence of chloramphenicol (30 µg/mL) and continued to grow at 37 °C. At 2 hours post re-inoculation, 1 mM Isopropyl-β-D-1-thiogalactopyranoside (IPTG) was added to the cultures and 20 mL sample was collected from each (control samples). The cultures were continued to grow at 37 °C and 8 hours after IPTG perturbation (10 hours post re-inoculation) another 20 mL culture was collected from each biological replicate (IPTG treated samples). Both control and IPTG treated samples were washed with PBS buffer 4 times and re-suspended in 8 M urea and 50 mM ammonium bicarbonate buffer. Cell lysis was performed with sonication (6 × 15 s, output level 2, 40%, 45 s on ice with a Sonifier 250 (Branson)). The soluble proteins were collected after centrifugation and quantified using a modified Bradford protein assay (BioRad). One hundred micrograms of protein from each condition was digested with trypsin and desalted with a Sep-Pak C18 desalting column (Waters) prior to mass spectrometry data acquisition. Three biological replicates from each condition and three technical replicates from each sample (total 18 MS runs) were acquired in DIA-MS mode and analyzed with Spectronaut (Biognosys) as described above.

## Data Records

### Data record 1

The mass spectrometry discovery DDA-MS proteomics data including instrument raw files (.wiff), converted mzML files (.mzML), identified peptides in the pepXML results (.pepxml) along with the fasta database (.fasta) that were used to generate the assay library, associated MAYU report (.tsv) and the spectral assay libraries (.txt) and its DIALib-QC reports (.tsv) have been deposited to the ProteomeXchange Consortium via the PRIDE^48^ partner repository with the dataset identifier, PXD020785^33^. The spectral assay libraries and their DIALib-QC reports are also available at www.swathatlas.org, linked to PXD020785^33^.

### Data record 2

The mass spectrometry SWATH-MS data including instrument raw files (.wiff) and HTRMS files (.htrms) with identified peptides and proteins as Spectronaut reports (.csv) used to compare the robustness in identification and consistency in quantitation of different chromatography gradient lengths and to report the quantitative analysis of IPTG treatment (.xlsx) have been deposited to the ProteomeXchange Consortium via the PRIDE^48^ partner repository with the dataset identifier PXD020761^49^.

## Technical Validation

### Generation and validation of a comprehensive high-quality *E. coli* DIA/SWATH spectral assay library

**Fig. 2:**
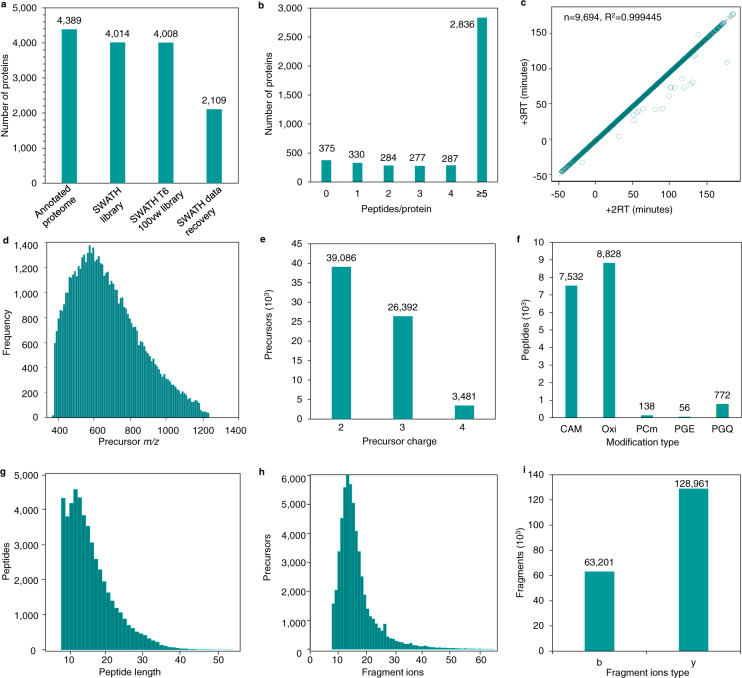
Coverage and characteristics of the *E. coli* spectral assay library. (**a)** Proteome coverage of the *E. coli* spectral assay library (complete library and library with top 6 ions and 100 variable windows applied) and SWATH-MS identified proteins with the developed library in comparison to the annotated reference proteome. (**b)** The graph depicts the number of *E. coli* peptides per protein in the SWATH assay library. (**c)** Retention time (RT) fit of +2 and +3 charge states of the same peptide in the assay library by DIALib-QC to assess the quality of the library. (**d)** Distribution of precursor *m/z* values across the acquired mass range in the assay library. (**e)** Frequency of precursor charge states observed in the assay library. (**f)** Frequency and type of peptide modification observed in the assay library. CAM: carbamidomethylation, Oxi: oxidation, PCm: S-carbamoylmethylcysteine, PGQ: pyroglutamate, and PGE: pyroglutamatic acid. (**g)** Distribution of peptide length in the assay library. (**h)** Distribution of the number of fragment ions per precursor. (**i)** Frequency of observed b- and y- ion fragments with CID fragmentation in the assay library.

Accurate identification and precise quantification of peptides and proteins with targeted DIA/SWATH analysis require validated high-quality assay libraries^17^. To generate a spectral assay library for *E. coli*, the DDA-MS datasets described above (see Methods and Data Records) were searched against the non-redundant, canonical *E. coli* reference proteome (UniProtKB/Swiss-Prot)^42^ and analyzed with the Trans-Proteomic Pipeline^38^, a standardized suite of software tools for the processing and analysis of MS/MS data **(**Fig. 1**)**. To avoid the accumulation of false positives both at the peptide and protein level, we applied MAYU^31^, which reliably estimates false discovery rates in large data sets, and adjusted the spectral assay library to an FDR of 1% at the protein level. This resulted in a comprehensive, high-quality spectral assay library with 802,083 transitions identifying 68,121 peptide precursors that represent 56,182 modified peptides, 48,188 stripped peptides and 4,014 proteins (91.5% of 4,389 annotated *E. coli* proteins) **(**Table 2, Fig. 2a and Supplementary Table 3**)**. While 99% of the peptides in the developed assay library are proteotypic, the library includes 690 peptides that are shared by different proteins, and both proteotypic and shared peptides combined support the identification of total 4,086 proteins (93.1% of the annotated proteome) or 4,151 protein groups. Applying a 100 variable window acquisition scheme for increased specificity, removing fragment ions that fall into the SWATH window of the precursor (Methods), and keeping the library to top 6 fragment ions, it covers 4,008 proteins **(**Fig. 2a**)**. The contribution of unique proteins from each sample type in the spectral assay library is summarized in Supplementary Note 1.

**Table 2:** Library statistics.

	Proteotypic	Proteotypic and Shared
**Proteins**	4,014	4,086
**Stripped Peptides**	48,188	48,771
**Modified Peptides**	56,182	56,872
**Precursors**	68,121	68,948
**Transitions**	802,083	811,406

Overview of the number of proteins, stripped peptides, modified peptides, precursor ions and transitions at 1% protein FDR for proteotypic peptides and all peptides (proteotypic and shared) in the assay library.

While we selected 1,358 synthetic peptides to provide assays for proteins that are difficult to detect (e.g., proteins that are only expressed under certain conditions), 82% of the proteins in the library are derived from cell lysate and expressed proteins, synthetic peptides make less than 3% of the peptides in the *E. coli* assay library. We then assessed the coverage of the *E. coli* assay library in terms of peptides per protein (Fig. 2b). While 330 proteins (8% of the proteome) were observed by only one peptide, 77% of the *E. coli* proteome (3,400 proteins) are represented by at least three peptides per protein and 65% (2,836 proteins) by five or more distinct peptides, demonstrating a deep proteome coverage of the *E. coli* proteome by the spectral assay library.

Comparing the extensive in-depth proteome coverage of 91.5% of the *E. coli* spectral library with DIA libraries from other bacterial species^10,50–52^ (available at www.swathatlas.org, except for *M. catarrhalis*^52^), we observed that their proteome coverage ranges from 36–96% **(**Supplementary Fig. 1**)**. The *M. tuberculosis* library^10^ also includes synthetic peptides and is the only library that provides with 96% an equally high proteome coverage as the *E. coli* spectral assay library presented here.

Next, we evaluated the quality and characteristics of the library with DIALib-QC to ensure correct identification and precise quantification of peptides and proteins during DIA-MS data analysis. DIALib-QC assesses the quality of the assay library by estimating the retention time (RT) fit of +2 and +3 charge states of the same peptide which reflects the quality of chromatography and retention time normalization based on reference peptides in the library. The DIALib-QC assessment reports >0.99 RT correlation (r^2^ value), signifying high RT similarity between +2 and +3 charge states of a same peptide **(**Fig. 2c and Supplementary Table 3**)**. In addition, DIALib-QC computes the difference in q3 *m/z* values between the experimental library and the theoretical mass values for each fragment ion. Small *m/z* differences have profound effects on peptide and protein identifications in DIA/SWATH analyses, no mass differences were observed, confirming theoretical q3 *m/z* values were used to generate the *E. coli* assay library **(**Supplementary Table 3).

In addition, several other features of the assay library were evaluated including its characteristics, complexity and modifications. The library covers a precursor mass range of 400 to 1,250 *m/z* **(**Fig. 2d**)** and peptide precursors are primarily of charge state two (53%), three (41%) and four (6%) **(**Fig. 2e). Reviewing the different types and frequency of modifications in the assay library, the largest group comprises 7,532 carbamidomethyled (CAM) (+57.0214 Da) peptides, a modification of cysteine residues introduced by the alkylation step using iodoacetamide to avoid the formation of disulfide bonds during sample preparation, and 8,828 oxidized (Oxi) (+15.9949 Da) peptides as methionine and tryptophan are prone to oxidation during sample preparation. We also observed 772 peptides with the cyclized modification of N-terminal glutamine residues referred to as pyroglutamate (PGQ) (−17.0 Da), 56 peptides with cyclized modification of N-terminal glutamic acid residues (PGE) (−17.0 Da) and 138 peptides with S-carbamoylmethylcysteine cyclization at the N-terminus (PCm) (+39.994915) **(**Fig. 2f). Peptide lengths ranged from 7 to 49 amino acids, with 98% of the total between 7 to 30 amino acids in length **(**Fig. 2g). The majority of peptides (>80%) have more than 10 fragment ions per precursor ensuring an adequate number of ions to estimate peptide quantities (Fig. 2h**)**. As expected, we observed a higher number of y than b fragment ions, 70% vs 30% respectively, with collision induced dissociation (CID) fragmentation **(**Fig. 2i**)**.

### Performance of the *E. coli* spectral assay library with different gradients by DIA/SWATH-MS analysis

**Fig. 3:**
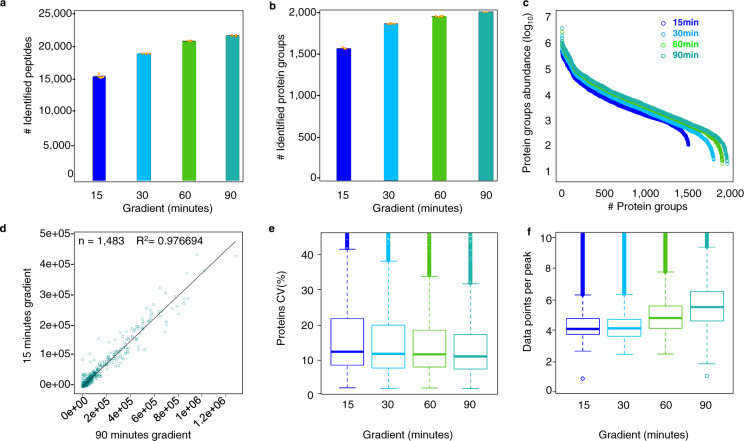
Performance of the spectral assay library with different liquid chromatography gradients by DIA/SWATH-MS. (**a)** Number of unique peptides and (**b)** protein groups identified with chromatography gradients of different length. An approximately 20% increase in peptide and protein group identifications was observed in the 90 minutes gradient compared to the 15 minutes gradient length. The error bars indicate the variability within five replicates represented as standard error of the mean. These are calculated as the ratio of standard deviation of the number of quantified peptides or proteins observed in each gradient replicate to the square-root of the sample size (n = 5). The small yellow dots denote the number of identifications in each replicate. (**c)** The plot shows all identified protein groups ranked according to their abundance, highlighting the dynamic range of proteins that can be quantified with liquid chromatography of different gradient length. All gradients resulted in protein quantification across five orders of magnitude, with exception of the 15 min gradient which covered four orders of magnitude. (**d)** Pearson correlation of protein intensity values obtained from 1,483 proteotypic proteins that were quantified in all five technical replicates by both, 15 minutes and 90 minutes gradients. The high positive correlation indicates quantitative robustness between the gradient methods. (**e)** Distribution of the coefficient of variation (CV) of proteins identified in all five replicates at 1% protein FDR estimated by Spectronaut. The median CV of 10% (90 minutes gradient) to 11% (15 minutes gradient) correlates well with the expected technical variation. The first and third quartile are marked by a box with whisker marking a minimum/maximum value ranging to 12 interquartile and the median depicted as solid line. (**f)** Distribution of data points per elution peak for the different gradient methods estimated by Spectronaut. The first and third quartile are marked by a box with whisker marking a minimum/maximum value ranging to 3 interquartile and the median depicted as solid line.

We measured *E. coli* whole cell lysate in DIA-MS mode with four different gradients to demonstrate the performance of the library (see Methods and Supplementary Fig. 2**)**. Precisely, we evaluated the effects of LC gradient lengths of 15, 30, 60, and 90 minutes regarding the identification and relative quantification of peptides and proteins. DIA-MS analysis with Spectronaut software resulted in the identification of 15,171 to 21,488 peptides and 1,558 to 2,003 protein groups at <1% protein FDR **(**Fig. 3a,b**)**. Among these identifications, 65 proteotypic peptides were detected with assays developed from synthetic peptide spectra. An example is peptide MQDLSLEAR.2 **(**Supplementary Fig. 3**)** demonstrating that peptides from synthetic peptide assays can be detected in WCL and increase the number of detected peptides per protein. Spectronaut determines the ideal XIC RT extraction windows dynamically for each analysis to support correct peak detection in gradients of different length **(**Supplementary Fig. 4**)**. With shorter gradient length a loss of sensitivity in terms of identifications was observed when compared to the other gradient profiles, whereas, as expected, the highest sensitivity with the largest dynamic range was achieved with the 90 minutes gradient **(**Fig. 3c**)**. However, relative to the 90 minutes gradient, we were able to quantify ~70% of the peptides and ~80% of the protein groups with the shortest 15 minutes gradient, which consumed only 27% of instrument time **(**Fig. 3a,b**)**. Only minimal losses in identification were observed with the 60 minutes gradient while still saving 28% acquisition time. We conclude from these observations that a higher volume of samples can be measured in a given time with a single short micro-LC gradient for the separation of peptides, thereby enabling the testing of multiple experimental conditions with complex proteomic samples such as *E. coli* total cell lysates.

Next, we evaluated robustness and reproducibility of quantitative measurements by comparing the protein abundances of the 15- and 90-minutes gradient method estimated by Spectronaut. 1,483 proteins were quantified by both methods with high positive correlation (r^2^ = 0.97) highlighting the consistency in quantification **(**Fig. 3d**)**. For all other gradient comparisons (15 vs. 30 minutes, 15 vs. 60 minutes, etc.), a minimum relative quantitation correlation of >0.98 was observed **(**Supplementary Fig. 5**)**, assuring the reproducibility in quantitation. Subsequently, we evaluated and compared the quantitative precision among different gradient methods by estimating the coefficient of variation (CV) for the obtained protein quantities. The median CVs of the proteins quantified in five technical replicates were 10–11%, similar for all gradient methods **(**Fig. 3e**)**. In addition, we assessed the number of data points measured across the elution profiles for precursors identified in all gradients using the SCIEX 6600 TripleTOF instrument. A median value of 4–6 data points per peak is obtained that allows optimal quantification of identified peptides using Spectronaut analysis **(**Fig. 3f and Supplementary Fig. 6a**)**. Here, we achieved more data points (4 + median data points per peak) with a 15 minutes HPLC gradient and the TripleTOF 6600 compared to recently reported DIA studies based on the Thermo-Fisher QE HFX (3 median Data points per peak)^53^ or Thermo-Fisher Exploris (3 + points per peak)^54^ instruments using similar gradient length and DIA settings. The effect of different gradient lengths on the base peak widths is exemplified with peptide IVSYAQGFSQLR in Supplementary Fig. 6b. These results demonstrate that the *E. coli* spectral assay library can be applied to fast chromatography methods to increase the sample throughput of proteomic analysis, without losing the reliability in identification and quantification of peptides and proteins.

### Quantitative analysis of differentially expressed IPTG treated proteins using the *E. coli* spectral assay library in DIA/SWATH-MS

To demonstrate the application of our comprehensive *E. coli* spectral library in a quantitative experiment, we studied the *E. coli* proteome during growth at two conditions. We used *E. coli* at log phase (2 hours post re-inoculation) as a control and compared it to a sample of the same growth collected at 8 hours with a perturbation with IPTG (10 hours post re-inoculation, IPTG treated). Proteins identified in three biological replicates (with three technical replicates each) from both conditions were compared. An average of 2,050 and 2,101 proteins were identified in the control (at 2 hours) and IPTG treated sample (at 10 hours), respectively **(**Supplementary Fig. 7a**)**. Next, we evaluated the reproducibility of measured protein abundances between two biological replicates (one and two) of control and IPTG samples. 1,902 proteins were quantified by both biological replicates for the control and 1,959 for both IPTG samples at a high positive correlation of r^2^ = 0.992 and r^2^ = 0.9848, respectively, indicating the consistency in quantification **(**Supplementary Fig. 7b**)**. Subsequently, we performed unsupervised hierarchical clustering on three biological replicates per condition. As expected, the biological replicates from a single condition were grouped together with similar expression profiles indicating quantitative reproducibility while the two conditions were clearly separated into different clusters based on their different protein expression profiles **(**Supplementary Fig. 7c**)**. Further, we depicted significant differentially expressed proteins between the two conditions in a volcano plot by applying a threshold of – log10 P‐value of less than 3 (p-value < 0.001) and log2 fold change of ≥+1 (up-regulated) or ≤−1 (down-regulated) **(**Supplementary Fig. 7d**)**. This resulted in 485 significantly differential expressed proteins, 291 up-regulated (red dots) and 194 down-regulated (green dots) proteins in the IPTG treated sample compared to the control. A significantly down-regulated protein is the DNA-binding protein Fis (UniProt P0A6R3) which plays an important role in DNA metabolism, chromosome replication and repair mechanisms^55^. The effect of prolonged IPTG treatment has resulted in its decreased expression by nearly 10-fold compared to the control sample as exemplified with a quantifying peptide AALMMGINR in Supplementary Fig. 7e. IPTG induction has been reported to generate metabolic stress and toxicity leading to a negative effect on the cellular growth of *E. coli*^56^. An example of an up-regulated protein is Sulfate adenylyltransferase subunit 1 (cysN) (UniProt P23845), a component of the enzyme ATP sulfurylase, which is known to play an important role in sulfur metabolism in *E. coli*^57,58^ **(**Supplementary Fig. 7f). It forms adenosine 5′-phosphosulfate (APS), an activated sulphate form that is a building block for sulfur-containing amino acids (Cysteine and Methionine) and 3′-phosphoadenosine 5′-phosphosulfate (PAPS)^57,59^. With the extended IPTG induction, the IPTG treated sample showed an increased protein expression of cysN to produce large quantities of these sulfur-containing amino acids for the pressure of increased protein translation^59^.

## Usage Notes

### Generation of SWATH validated spectral libraries from full spectral library

In this study, we constructed an *E. coli* spectral assay library and extracted a SWATH validated library by applying a 100 variable window isolation scheme, and by containing the six most intense fragments ions, the library is named “*E. coli* SWATH T6 100vw” library. However, the full *E. coli* spectral assay library can be validated and assessed with any other acquisition window scheme or configuration using spectrast2tsv.pv and DIALib-QC as described in the method section.

### Compatibility with different chromatography and gradient length setups

We evaluated the performance of the *E. coli* SWATH library by measuring the samples using a micro-flow single-shot short gradient DIA/SWATH-MS method intended to accelerate the discovery, verification and quantitation of *E. coli* proteins. In addition, the generated *E. coli* SWATH library can be used for the analysis of data acquired with different chromatography flow rates, such as nano-flow, and with methods of different gradient lengths. Since the *E. coli* SWATH library RT is in iRT space, users can spike-in iRT reference peptides^46^ into samples to normalize the retention time for targeted DIA/SWATH data analysis.

### Compatibility with most commonly used software for peptide centric DIA/SWATH analysis

In this study, we provide both PeakView and OpenSWATH formats of the *E. coli* SWATH spectral assay libraries. The current SWATH-MS analysis was performed by importing the assay library in PeakView format into the Spectronaut analysis software. The PeakView format of the library can be used with PeakView, Spectronaut and Skyline software as per their recommended data analysis workflows and the OpenSWATH format can be used with the OpenSWATH workflow to analyze DIA/SWATH data.

### Spectral assay library portability

The high-quality assay library of the *E. coli* proteome is a portable resource that can be used for the analysis of SWATH data generated on SCIEX TripleTOF platforms. To demonstrate its application on DIA data collected on a Thermo instrument, we compared the performance using a sample specific Orbitrap Fusion BW25113 *E. coli* library^60^ and our TripleTOF spectral library. The TripleTOF *E. coli* spectral library comprises additional 70% peptides and 50% proteins that are exclusive when compared to the sample specific Orbitrap Fusion library which only adds 3% unique peptides and 0.5% proteins **(**Supplementary Fig. 8a,b**)**. In the comparative DIA analysis, the TripleTOF assay library resulted in the identification of a higher number of peptides and proteins than its counterpart library **(**Supplementary Fig. 8c,d**)**. This analysis concludes that our *E. coli* library generated from data acquired on a TripleTOF instrument can be used to analyze data collected on other instruments such as an Orbitrap Fusion utilizing HCD fragmentation and that it is superior to its counterpart library derived from the Orbitrap Fusion HCD data when using the same Orbitrap Fusion DIA raw data.

## Supplementary information

Supplementary Information
