# Supplementary material for: A comprehensive spectral assay library to quantify the Escherichia coli proteome by DIA/SWATH-MS
Source: Sci Data. 2020 Nov 12;7:389. doi: 10.1038/s41597-020-00724-7 (PMC7665006; doi:10.1038/s41597-020-00724-7)
Supplement: Supplementary file 1 — Supplementary Information [file 41597_2020_724_MOESM1_ESM.pdf]

SUPPLEMENTARY INFORMATION

A comprehensive spectral assay library to quantify the *Escherichia coli* proteome by DIA/SWATH-MS

Mukul K. Midha[1], Ulrike Kusebauch[1], David Shteynberg[1], Charu Kapil[1], Samuel L. Bader[1], Panga Jaipal Reddy[1], David S. Campbell[1], Nitin S. Baliga[1,2,3,4] and Robert L. Moritz[1*]

[1]Institute for Systems Biology, 401 Terry Ave N, Seattle, WA, 98109, USA

[2] Departments of Biology and Microbiology, University of Washington, Seattle, WA, USA

[3] Molecular and Cellular Biology Program, University of Washington, Seattle, WA, USA

[4] Lawrence Berkeley National Lab, Berkeley, CA, USA

*Address correspondence to: Robert L. Moritz, Institute for Systems Biology, 401 Terry Ave N, Seattle, WA 98109, USA, Email: rmoritz@systemsbiology.org, Phone: 206-732-1200

Table of Contents

Supplementary Figure 1. Proteome coverage of DIA spectral libraries of different bacterial species. 3

Supplementary Figure 2. Overlay of total ion chromatograms (TIC) for five SWATH-MS runs of different gradient lengths. 4

Supplementary Figure 3. Detection of endogenous peptide MQDLSLEAR.2 with synthetic peptide derived assay in Spectronaut analysis 5

Supplementary Figure 4. Extracted Ion Chromatograms (XIC) width plots. 6

Supplementary Figure 5. Scatter plots showing Pearson correlation of protein intensity values in different gradients. 7

Supplementary Figure 6. Distribution of data points per peak in five SWATH replicates across different gradient methods and their effect on the peak width in Spectronaut analysis. 8

Supplementary Figure 7. Analysis of differentially expressed proteins using the *E. coli* spectral assay library in DIA/SWATH-MS. 9

Supplementary Figure 8. Comparative analysis of the performance of Orbitrap Fusion DIA-MS data using the TripleTOF *E. coli* spectral assay library. 12

Supplementary Table 1. Gradients used for the separation of *E. coli* peptides in DDA-MS. 13

Supplementary Table 2. Gradients used for the separation of *E. coli* peptides in DIA/SWATH-MS mode. 14

Supplementary Table 3. DIALib-QC assessment report for the *E. coli* SWATH spectral assay libraries of PeakView and OpenSWATH formats. 15

Supplementary Note 1: Contribution of proteins per sample type in the spectral assay library 16

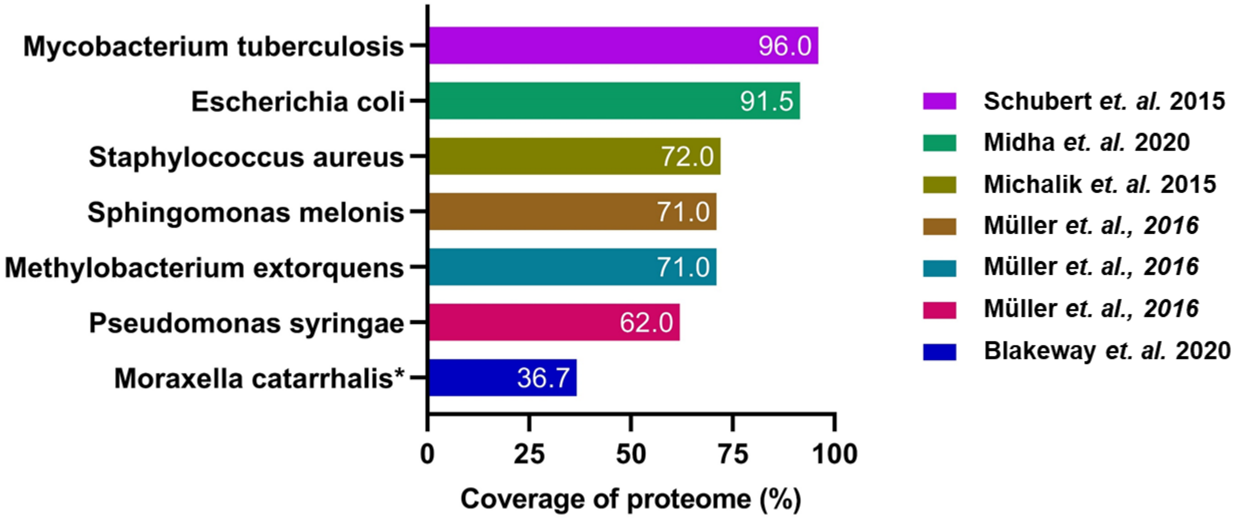

Supplementary Figure 1. Proteome coverage of DIA spectral libraries of different bacterial species. The proteome coverage of *M. tuberculosis*[1], *S. aureus*[2], *Sphingomonas melonis*[3], *Methylobacterium extorquens*[3] and *Pseudomonas syringae*[3] was obtained from the respective publication, and for *M. catarrhalis*[4] (marked with an asterisk) it was calculated using the number of protein entries in the spectral library against the total number of proteins in the sequence database.

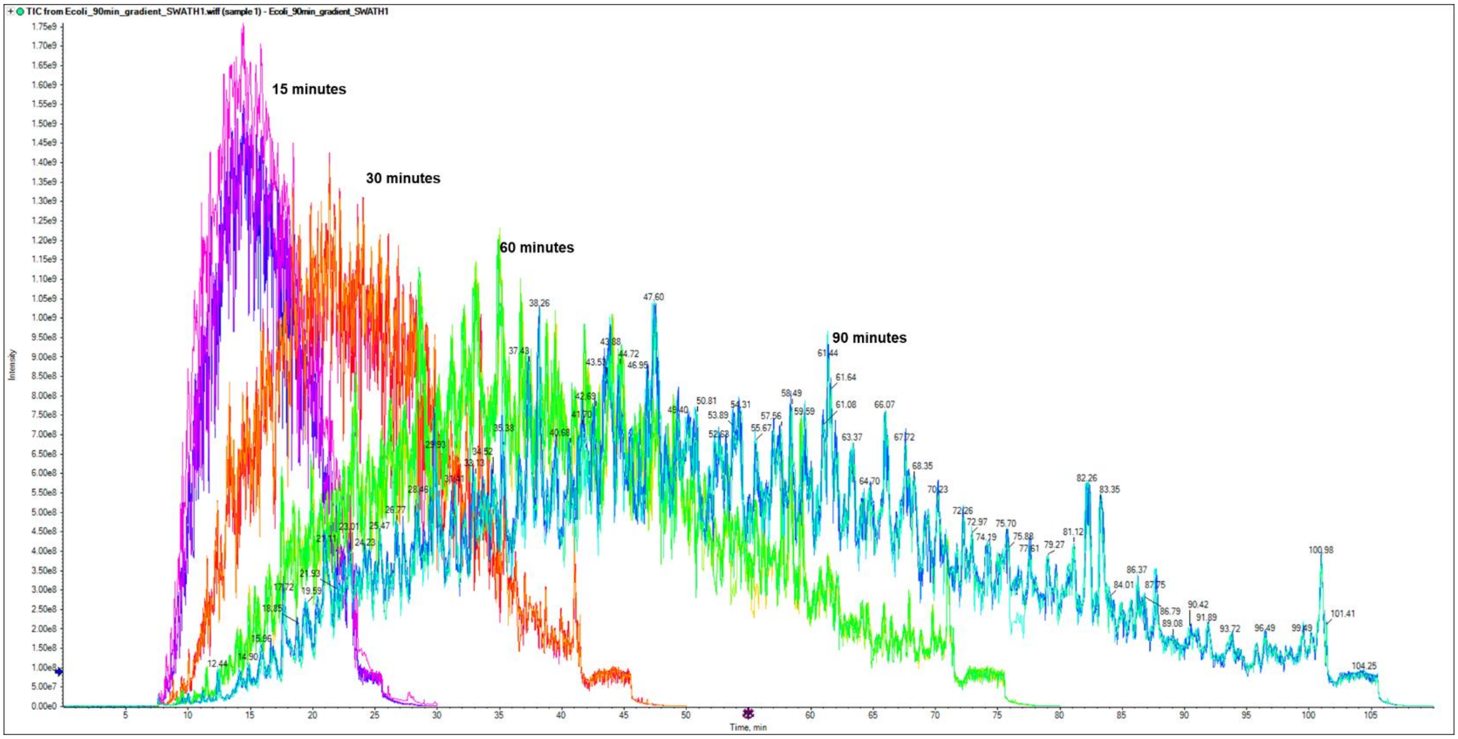

Supplementary Figure 2. Overlay of total ion chromatograms (TIC) for five SWATH-MS runs of different gradient lengths. Purple, red, green and blue colored TIC represents 15, 30, 60, and 90 minutes gradient lengths respectively.

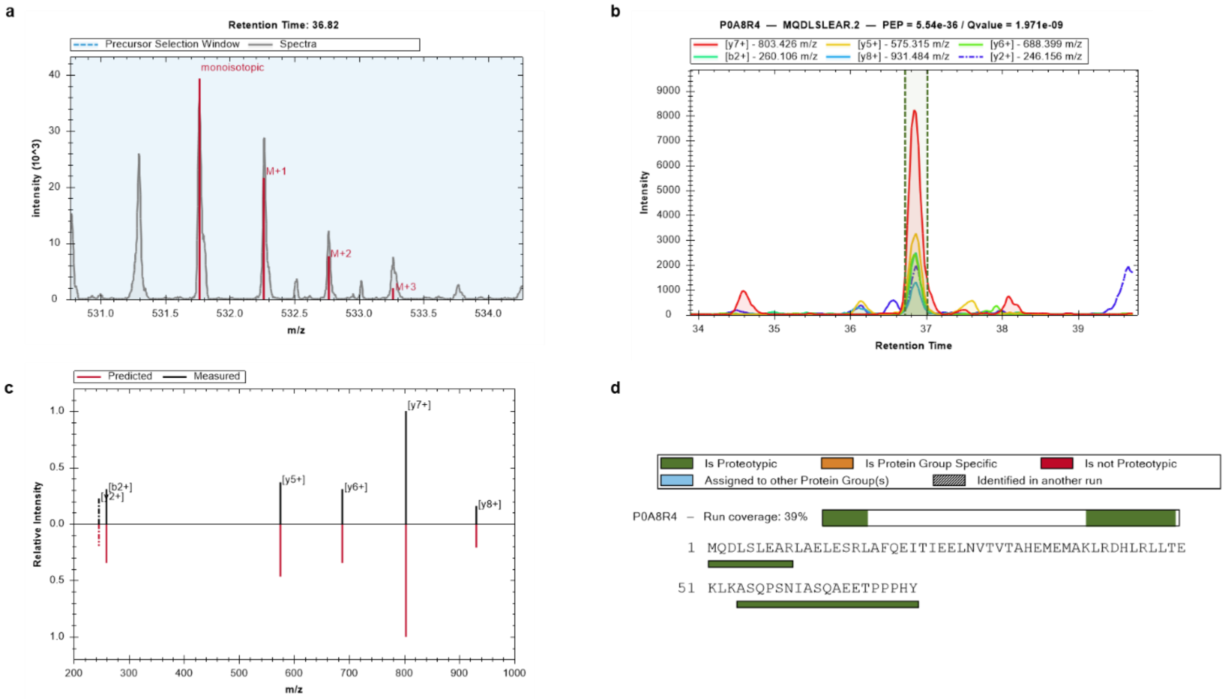

Supplementary Figure 3. Detection of endogenous peptide MQDLSLEAR.2 with synthetic peptide derived assay in Spectronaut analysis. a) MS1 spectrum at the apex with retention time 36.82 minutes. **b)** MS2 analysis based on extracted ion chromatogram (XIC). **c)** Correlation plot of measured (solid black, DIA data) and expected (solid red, library) relative fragment ion intensities. Five well correlating fragment ions are depicted as solid lines, the unassigned fragment ion is represented as dotted line. **d)** Protein coverage plot for protein P0A8R4, 39% of the sequence are covered by proteotypic peptides identified at 0.01 q-value. The detected N-terminal peptide MQDLSLEAR is based on assays developed from synthetic peptides and increasing the coverage of protein P0A8R4 in the DIA analysis that otherwise would have been represented by only one peptide (C-terminal peptide ASQPSNIASQAEETPPPHY, assay derived from endogenous source).

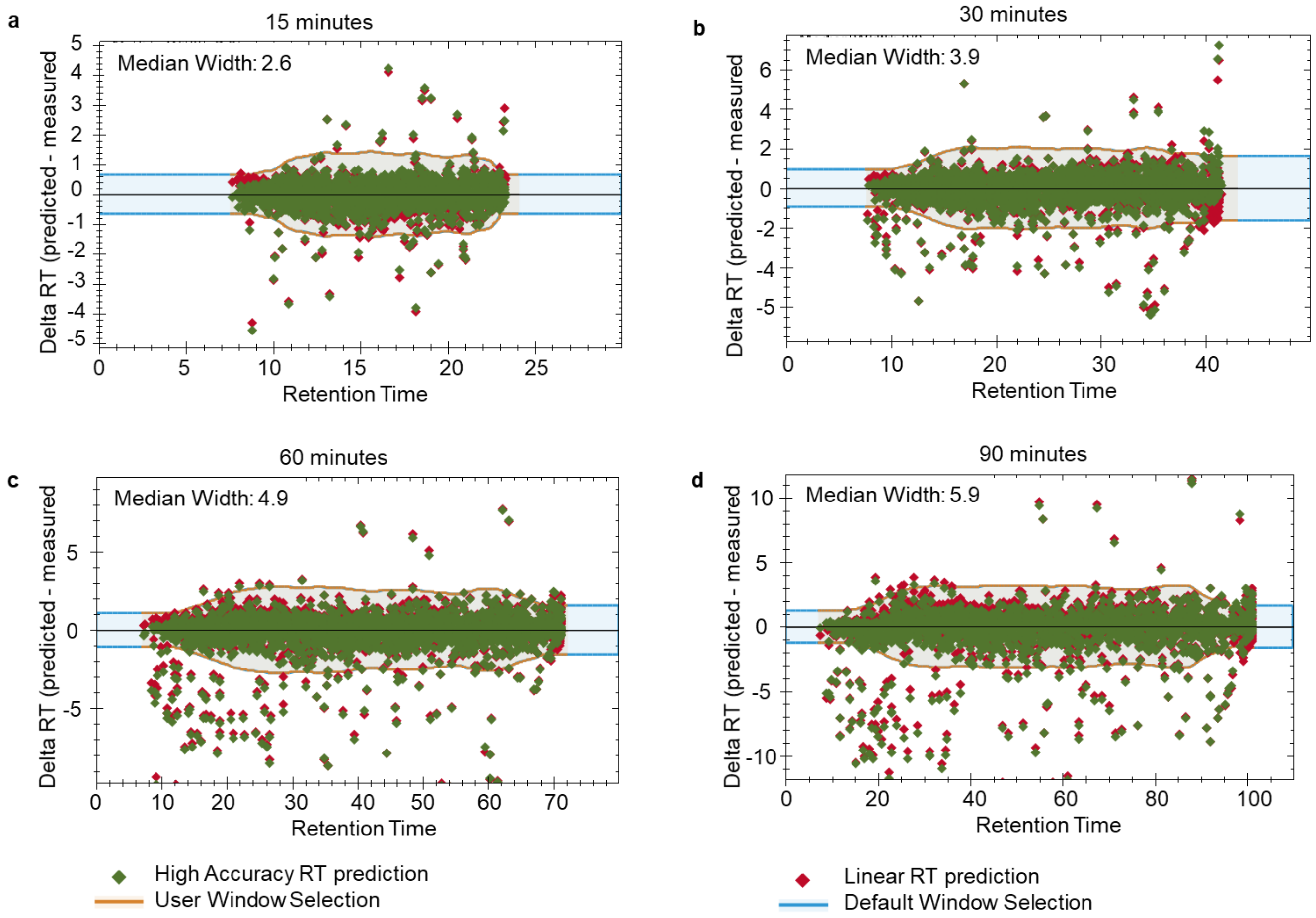

Supplementary Figure 4. Extracted Ion Chromatograms (XIC) width plots. The XIC extraction width plots depict different XIC widths, dynamically selected for each gradient by Spectronaut, and reflect gradient stability and overall accuracy of a library's iRT values. While the blue line indicates the default window selection, the orange line shows the user adjusted XIC width, which changes over time based on gradient stability and iRT accuracy. The red dots show the iRT accuracy assuming a linear iRT to RT transformation and the green dots the extended non-linear iRT to RT transformation that was applied in this analysis for the **a)** 15 minutes, **b)** 30 minutes, **c)** 60 minutes, and **d)** 90 minutes gradient.

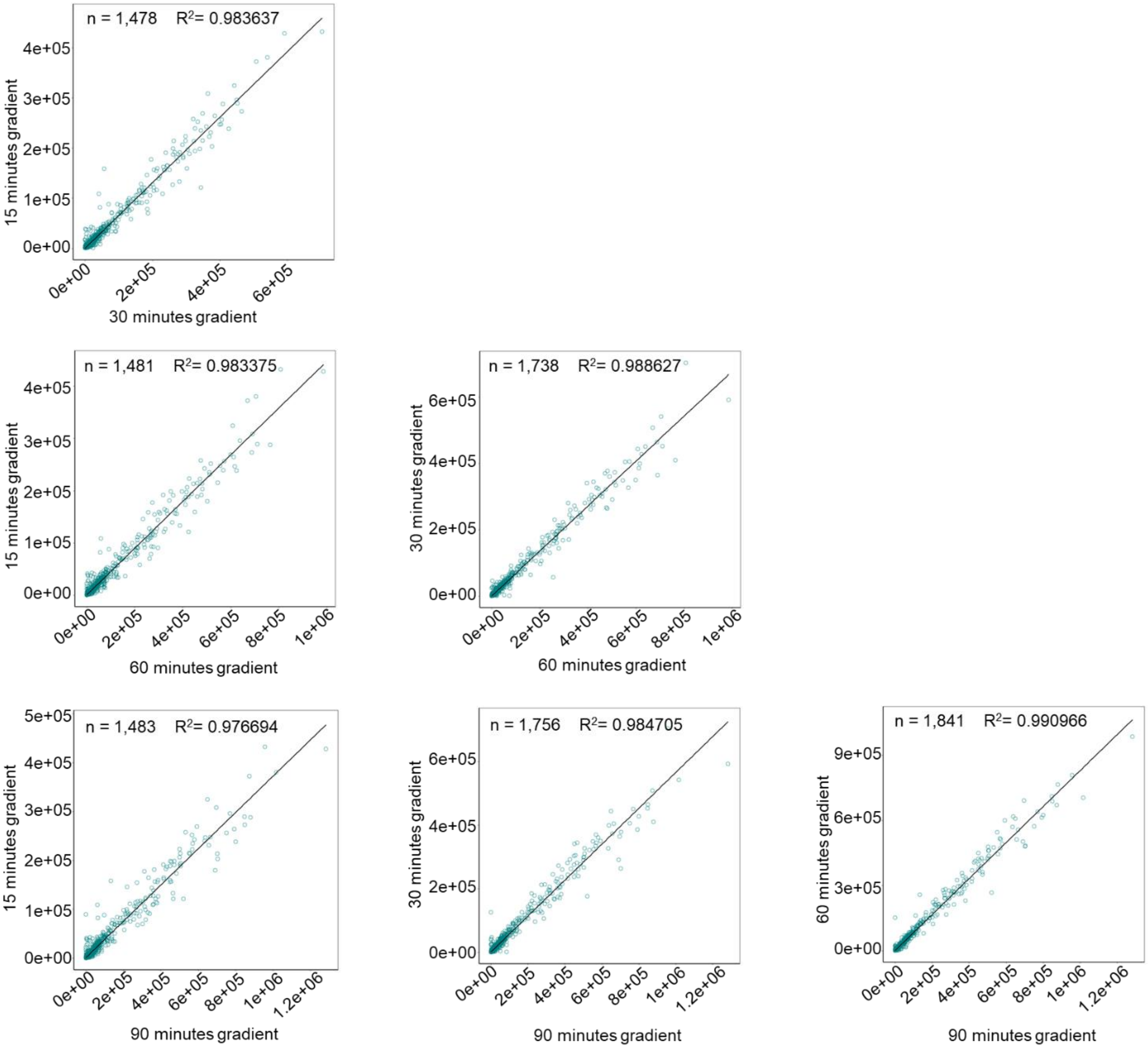

Supplementary Figure 5. Scatter plots showing Pearson correlation of protein intensity values in different gradients. The high positive correlation indicates robust quantitation was achieved using the *E. coli* SWATH assay library. All proteins considered for quantitation scatter plots were mapped with proteotypic peptides and were observed in all five SWATH technical replicates for all gradient methods.

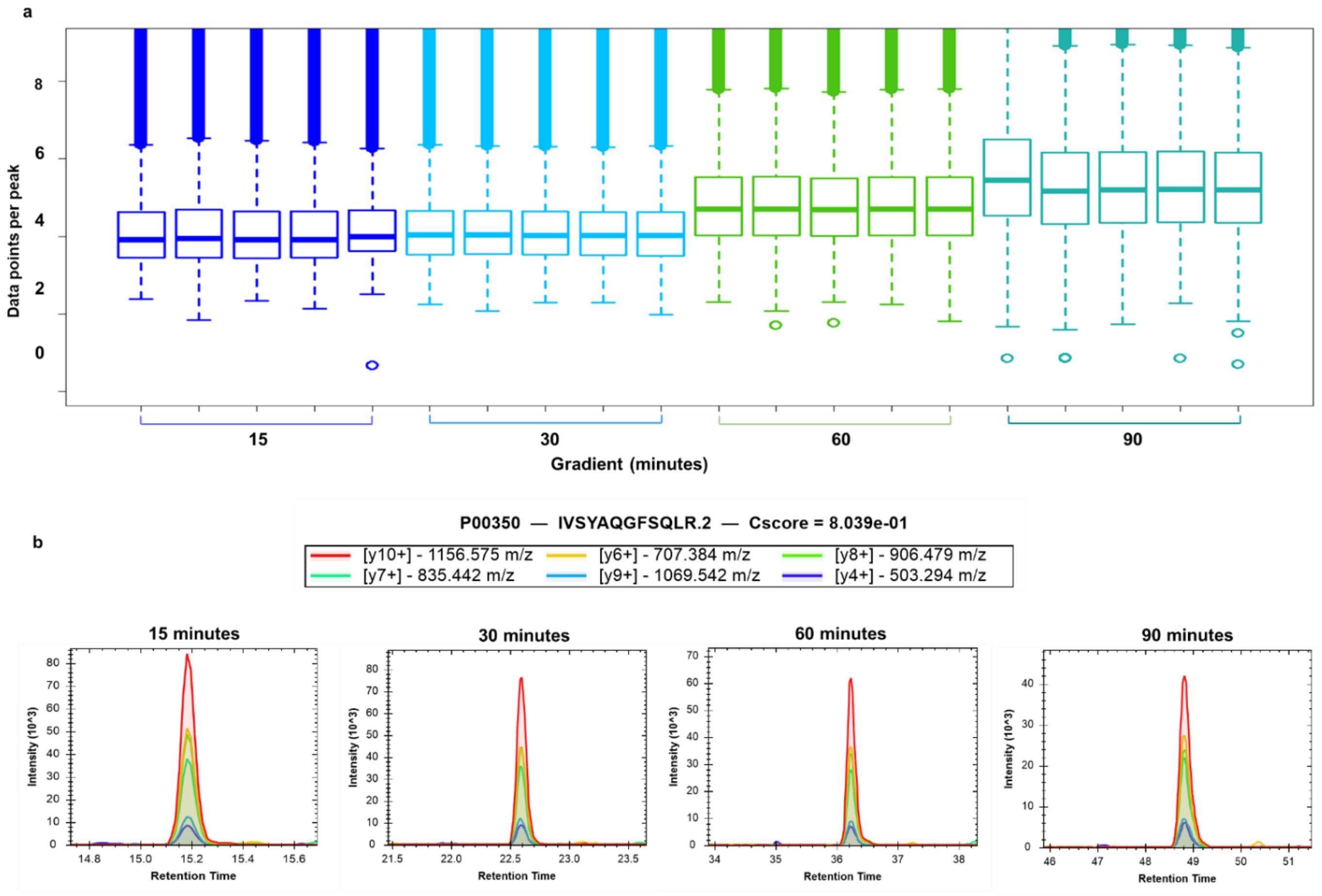

Supplementary Figure 6. Distribution of data points per peak in five SWATH replicates across different gradient methods and their effect on the peak width in Spectronaut analysis. a) For 100 variable precursor isolation windows, both the MS1 and MS2 accumulation time was adjusted for different gradient methods to achieve 4-6 data points per elution peak. The first and third quartile are marked by a box with whisker marking a minimum/maximum value ranging to 3 interquartile and a median highlighted as solid line. **b)** XIC plots of single peptide IVSYAQGFSQLR representing each gradient, highlights consistent chromatography peak widths per gradient (acetonitrile concentration) % change with increasing gradient lengths.

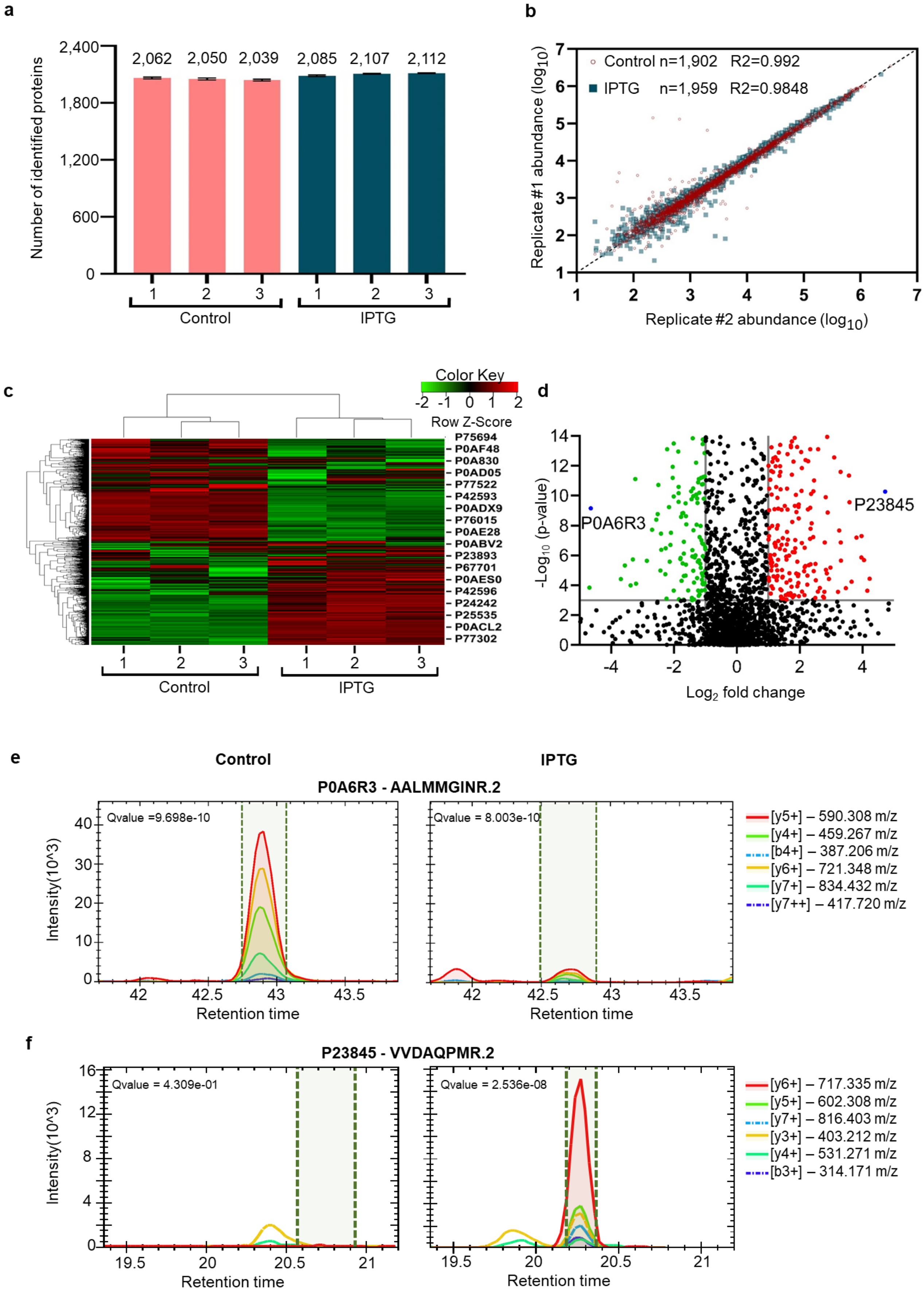
a
Number of identified proteins
2,400
1,800
1,200
600
0
2,062
2,050
2,039
2,085
2,107
2,112
1
2
3
1
2
3
Control
IPTG
b
Control n=1,902 R2=0.992
IPTG n=1,959 R2=0.9848
Replicate #1 abundance (log10)
Replicate #2 abundance (log10)
c
Color Key
-2 -1 0 1 2
Row Z-Score
P75694
P0AF48
P0A830
P0AD05
P77522
P42593
P0ADX9
P76015
P0AE28
P0ABV2
P23893
P67701
P0AES0
P42596
P24242
P25535
P0ACL2
P77302
1
2
3
1
2
3
Control
IPTG
d
-Log10 (p-value)
P0A6R3
P23845
Log2 fold change
e
Control
IPTG
P0A6R3 - AALMMGINR.2
Qvalue =9.698e-10
Qvalue = 8.003e-10
Intensity(10^3)
Retention time
Retention time
[y5+] – 590.308 m/z
[y4+] – 459.267 m/z
[b4+] – 387.206 m/z
[y6+] – 721.348 m/z
[y7+] – 834.432 m/z
[y7++] – 417.720 m/z
f
P23845 - VVDAQPMR.2
Qvalue = 4.309e-01
Qvalue = 2.536e-08
Intensity(10^3)
Retention time
Retention time
[y6+] – 717.335 m/z
[y5+] – 602.308 m/z
[y7+] – 816.403 m/z
[y3+] – 403.212 m/z
[y4+] – 531.271 m/z
[b3+] – 314.171 m/z

Supplementary Figure 7: Analysis of differentially expressed proteins using DIA/SWATH-MS and the *E. coli* spectral assay library. a) Comparison of protein groups identified in three biological replicates of control (2 hours post re-inoculation) and IPTG treated samples (8 hours-post 1mM IPTG). The standard error bars (mean) show variability within three technical replicates of each biological replicate for a condition. These are calculated as the ratio of standard deviation of the number of quantified proteins observed at each condition replicate to the square-root of the sample size (n=3). **b)** Pearson correlation of protein abundance values obtained from 1,902 (control) and 1,959 (IPTG treated) proteotypic proteins that were quantified in all three technical replicates of each biological replicate. **c)** Heatmap showing the hierarchical clustering of three biological replicates from two conditions (control and IPTG treated) of differentially expressed proteins (n=2,180). The relative protein abundance values for each sample were transformed to a logarithmic scale (log2), normalized and clustered based on the Euclidean distance and ward method linkage strategy. The columns in the heat map correspond to the three biological replicates per condition. The red and green color represents higher and lower relative protein abundances, respectively. **d)** Volcano plot of significant differentially expressed proteins between control and IPTG treated samples of *E. coli*. Proteins are separated according to the $\log_2$ fold change (x-axis) and the $-\log_{10}$ of the P-values based on a two-sample t-test (y-axis). A total of 485 significant differentially expressed proteins were found with increased (red dots) or decreased expression (green dots) in the IPTG treated compared to the control sample (P-value <0.001; fold change ≥ 2 or ≤ 0.5). For presentation purposes, we show only 293 such regulated proteins (limited to a cut off value of –log10 (p value) of 14). An example for a highly significant down-regulated and up-regulated protein is shown with P0A6R3 and P23845, respectively, labeled in blue color in

the volcano plot. The grey grid lines mark the values of 3 at the y-axis and -1 and 1 at the x-axis separating the significant and regulated expressed proteins from the complete data set. MS2-XIC plots of **e)** peptide AALMMGINR.2 mapped to DNA binding protein Fis (UniProt P0A6R3) highlights decreased signal intensity and **f)** peptide VVDAQPMR.2 mapped to Sulfate adenylyltransferase subunit 1 (UniProt P23845) highlights increased signal intensity, respectively, in the IPTG treated sample compared to the control sample.

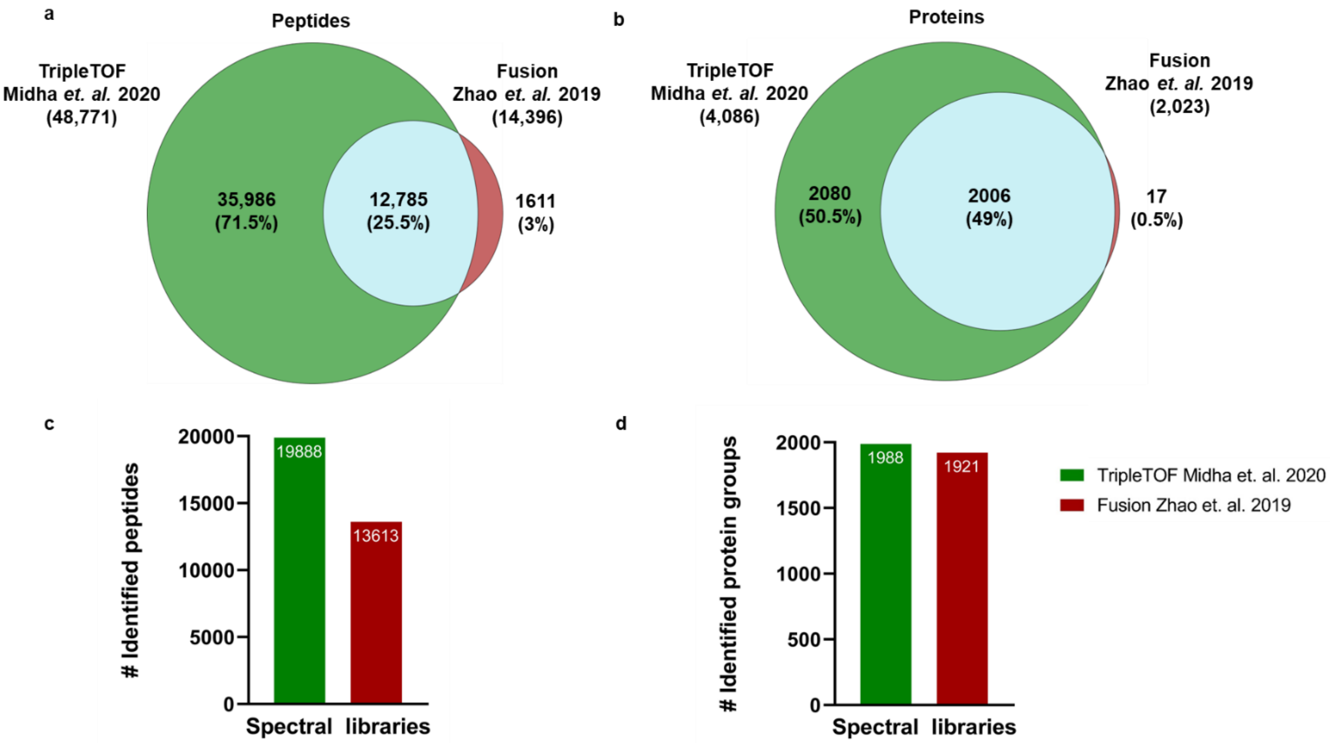

Supplementary Figure 8. Comparative analysis of the performance of Orbitrap Fusion DIA-MS data using the TripleTOF *E. coli* spectral assay library. Venn diagrams depicting the number and overlap of **a)** peptides and **b)** proteins between TripleTOF (Sciex) Midha *et al.* 2020 and Orbitrap Fusion (Thermo) Zhao *et al.* 2019 spectral libraries[5]. Comparing the performance of these libraries using DIA-MS raw data collected on an Orbitrap Fusion, the identification of **c)** peptides and **d)** proteins using the TripleTOF comprehensive library results in a higher number of identifications at both the peptide and protein level.

Supplementary Table 1. Gradients used for the separation of *E. coli* peptides in DDA-MS.

60-minute gradient		
Time (minutes)	%A	%B
0	97	3
1	97	3
60	65	35
63	20	80
73	20	80
76	97	3
96	97	3

90-minute gradient		
Time (minutes)	%A	%B
0	97	3
1	97	3
90	65	35
95	60	40
97	20	80
100	20	80
101	97	3
110	97	3

120-minute gradient		
Time (minutes)	%A	%B
0	97	3
1	97	3
120	65	35
123	20	80
133	20	80
136	97	3
156	97	3

Supplementary Table 2. Gradients used for the separation of *E. coli* peptides in DIA/SWATH-MS mode.

15-minute gradient		
Time (minutes)	%A	%B
0	97	3
15	65	35
17	60	40
19	20	80
20	20	80
21	97	3
30	97	3

30-minute gradient		
Time (minutes)	%A	%B
0	97	3
30	65	35
35	60	40
37	20	80
40	20	80
41	97	3
50	97	3

60-minute gradient		
Time (minutes)	%A	%B
0	97	3
60	65	35
65	60	40
67	20	80
70	20	80
71	97	3
80	97	3

90-minute gradient		
Time (minutes)	%A	%B
0	97	3
90	65	35
95	60	40
97	20	80
100	20	80
101	97	3
110	97	3

Supplementary Table 3. DIALib-QC assessment report for the *E. coli* SWATH spectral assay libraries of PeakView and OpenSWATH formats.

Attribute	Value	Value	Value	Definition
library	Ecoli_consensus_peakview	Ecoli_consensus_T6_100vw_peakview	Ecoli_consensus_openswath	Name of library file being analyzed
format	Peakview	Peakview	OpenSWATH	Library format, one of OpenSWATH, PeakView, or Spectronaut
pepions	68959	68117	68959	Number of peptide ions (i.e. precursor, sequence + modifications + charge)
fragments	811524	408702	811524	Number of fragment (fragment ions) in library
ptp_percent	98.8	98.8	98.8	Percentage of proteotypic pepions (not shared)
shared_percent	1.2	1.2	1.2	Percentage of shared peptide ions (pepions)
shared_pepions	827	819	827	Number of shared pepions
peptides	48782	48371	48782	Number of distinct peptide sequences
mod_peps	56883	56365	56883	Number of distinct modified peptides (sequences + modifications)
mod_percent	25.1	25.1	25.1	percentage of distinct modified peptides with a mass modification
total_mods	17323	17172	17323	Number of mass modified amino acids
chg_2	53.1	56.4	53.1	Percentage of charge 2 precursors
chg_3	41.4	38.5	41.4	Percentage of charge 3 precursors
precursor_min	399.7	399.7	399.7	Minimum precursor m/z (mass/charge) in library
precursor_max	1248.7	1248.7	1248.7	Maximum precursor m/z in library
fragment_min	100.1	100.1	100.1	Minimum fragment m/z in library
fragment_max	1500	1499.9	1500	Maximum fragment m/z in library
avg_len	15.2	15.2	15.2	Average peptide Length
avg_num_frags	11.77	6	11.77	Average number of fragments per assay (precursor)
avg_frag_len	0.77	0.39	0.77	Average fragment sequence length
short_perc	1	0	1	Percentage of assays with 5 or fewer transitions
fragment_above_precursor	0.508	0.577	0.508	Percentage of fragment m/z above precursor m/z
y_perc	70.2	77.6	70.2	Percentage of y ions
b_perc	29.8	22.4	29.8	Percentage of b ions
t6_y_perc	77.8	77.6	77.8	Percentage of y ions considering only the top 6 fragments per assay
avg_intensity	3386.9	5030.6	3386.9	Average Intensity
rt_min	-50.6	-50.6	-50.6	Minimum RT (retention time) in library
rt_max	196.9	196.9	196.9	Maximum RT in library
rt_med	49.1	49.2	49.1	Median RT in library
rt_rsq	1	1	1	r-squared value of fit between RT of +2 and +3 charge states for the same modified peptide
rt_five	100	100	100	Percentage of +2/+3 charge pairs of the same mod pep within 5 RT units of each other
n_irt	11	11	11	Number of iRT peptides in library
irt_cnt	119	72	119	Number of iRT assays (precursor + fragment)
low_nr	37407	26543	37407	Number of fragments annotated as y1,y2,b1, or b2
db_peps	148954	148954	148954	Peptides in reference library, often 7-50 AA
seen_peps	30401	30057	30401	Number of library peptides seen
mc_peps	10106	10058	10106	Number of Missed cleavage peptides
db_prots	8987	8987	8987	Number of Proteins in reference library
lib_prots	4087	4081	4087	Number of proteins with at least one assay in ion library
seen_prots	4087	4081	4087	Number of library proteins seen
ux_prots	0	0	0	Unexplained (not in reference library) proteins
decoy_pct	0	0	0	Percentage of decoy (optionally includes "alternative", non-db decoys) assays
mixed_pct	0	0	0	Percentage of mixed decoy/target (has both decoy and no-decoy annotations) assays
fwd_pct	100	100	100	Percentage of target (non-decoy) assays
med_ppp	11	10	11	Median number of pepions per protein
mean_ppp	17.1	17	17.1	Mean number of pepions per protein
stddev_ppp	22.1	21.9	22.1	Standard deviation of the number of pepions per protein
3_sigma_ppp	80	81	80	number of pepions per protein more than 3 standard deviations from the mean
max_intensity_idx	1	1	1	Average index of most intense fragment
precursor_ok	68959	68117	68959	Number of assays where precursor is within 5 PPM (parts per million m/z) of theoretical
precursor_bad	0	0	0	Number of assays where precursor is more than 5 PPM from theoretical
fragment_ok	811524	408702	811524	Number of assays where fragment is within 1 PPM of theoretical
fragment_bad	0	0	0	Number of assays where fragment is more than 1 PPM from theoretical
fragment_na	0	0	0	Number of assays where peak annotation not found in expected b/y series
fragment_avg_mdiff	0	0	0	Average m/z difference between reported and theoretical fragment
problem_assays	0	0	0	Assays whose precursor or any fragment m/z values do not match SWATHs file or differ significantly from theoretical values

Supplementary Note 1: **Contribution of unique proteins per sample type in the spectral assay library.** Among the three different sample types, whole cell lysate (WCL), ASKA and synthetic peptides (SP) and two fractionated (off-gel electrophoresis (OGE) and differential mobility separation (DMS)) WCL samples analyzed in the present study, we found that the overexpressed ASKA samples and selected SPs have collectively accounted for the highest number of unique identifications in the consensus assay library. In total, 774 (19%) and 531 (13%) proteotypic proteins were exclusively detected in ASKA and SP samples, respectively. In the assay library, at 1% protein FDR, DMS fractions contributed a total of 1,803 proteotypic proteins, out of which, three proteins were unique. DMS showed a high agreement of identified proteins with other samples, specifically with ASKA. The rationale of including DMS samples into the spectral library was primarily to take the advantage of this acquisition strategy in getting cleaner, less inferred MS/MS spectra[6] from complex WCL samples. In addition, the OGE fractions provided 173 (4%) unique proteotypic proteins to the spectral library.

1. Schubert, O. T. *et al.* Absolute proteome composition and dynamics during dormancy and resuscitation of mycobacterium tuberculosis. *Cell Host Microbe* **18**, 96–108 (2015).

2. Depke, M. *et al.* A peptide resource for the analysis of Staphylococcus aureus in host-pathogen interaction studies. *Proteomics* **15**, 3648–3661 (2015).

3. Muller, D. B., Schubert, O. T., Rost, H., Aebersold, R. & Vorholt, J. A. Systems-level proteomics of two ubiquitous leaf commensals reveals complementary adaptive traits for phyllosphere colonization. *Mol. Cell. Proteomics* **15**, 3256–3269 (2016).

4. Blakeway, L.V. *et al.* Proteome of a *Moraxella catarrhalis* Strain under Iron-

Restricted Conditions. *Microbiol Resour Announc* **9**:e00064-20 (2020).

5. Zhao, J. *et al.* Multifaceted stoichiometry control of bacterial operons revealed by deep proteome quantification. *Front. Genet.* **10**, 1–15 (2019).

6. Campbell, J. L., Le Blanc, J. Y. & Kibbey, R. G. Differential mobility spectrometry: A valuable technology for analyzing challenging biological samples. *Bioanalysis* **7**, 853–856 (2015).
